# Transposable elements that have recently been mobile in the human genome

**DOI:** 10.1186/s12864-021-08085-0

**Published:** 2021-11-03

**Authors:** Matias I. Autio, Talal Bin Amin, Arnaud Perrin, Jen Yi Wong, Roger S.-Y. Foo, Shyam Prabhakar

**Affiliations:** 1 Laboratory of Epigenomics and Chromatin Organization, Genome Institute of Singapore, A*STAR, Singapore, 138672 Singapore; 2 Cardiovascular Research Institute, Yong Loo Lin School of Medicine, National University of Singapore, Singapore, 117599 Singapore; 3 Spatial and Single Cell Systems, Genome Institute of Singapore, A*STAR, 60 Biopolis St, Genome #02-01, Singapore, 138672 Singapore

**Keywords:** Transposable element, Mobile element, Insertion, Polymorphism, Human

## Abstract

**Background:**

Transposable elements (TE) comprise nearly half of the human genome and their insertions have profound effects to human genetic diversification and as well as disease. Despite their abovementioned significance, there is no consensus on the TE subfamilies that remain active in the human genome. In this study, we therefore developed a novel statistical test for recently mobile subfamilies (RMSs), based on patterns of overlap with > 100,000 polymorphic indels.

**Results:**

Our analysis produced a catalogue of 20 high-confidence RMSs, which excludes many false positives in public databases. Intriguingly though, it includes HERV-K, an LTR subfamily previously thought to be extinct. The RMS catalogue is strongly enriched for contributions to germline genetic disorders (*P* = 1.1e-10), and thus constitutes a valuable resource for diagnosing disorders of unknown aetiology using targeted TE-insertion screens. Remarkably, RMSs are also highly enriched for somatic insertions in diverse cancers (*P* = 2.8e-17), thus indicating strong correlations between germline and somatic TE mobility. Using CRISPR/Cas9 deletion, we show that an RMS-derived polymorphic TE insertion increased the expression of *RPL17*, a gene associated with lower survival in liver cancer. More broadly, polymorphic TE insertions from RMSs were enriched near genes with allele-specific expression, suggesting widespread effects on gene regulation.

**Conclusions:**

By using a novel statistical test we have defined a catalogue of 20 recently mobile transposable element subfamilies. We illustrate the gene regulatory potential of RMS-derived polymorphic TE insertions, using CRISPR/Cas9 deletion in vitro on a specific candidate, as well as by genome wide analysis of allele-specific expression. Our study presents novel insights into TE mobility and regulatory potential and provides a key resource for human disease genetics and population history studies.

**Supplementary Information:**

The online version contains supplementary material available at 10.1186/s12864-021-08085-0.

## Background

Transposable elements (TEs) are DNA sequences that can alter their location in the genome. They are ubiquitous: ~ 48% of the human genome can be directly annotated as TE-derived [1]. TEs are of particular importance because they can modify or create genes and gene families [2–4]. Moreover, insertion of new TE copies into the genome frequently imposes a fitness cost, which results in an evolutionary arms race between active TE families and host factors that evolve to silence them [5, 6]. TEs have also been shown to modulate the expression nearby genes by acting as cis-regulatory elements (promoters, enhancers or repressors) [7–11]. In addition, they can contribute to numerous diseases through insertional mutagenesis by disrupting coding sequences or splicing [12–15] and developmental disorders [16]. Finally, there is substantial evidence that somatic TE insertion can upregulate oncogenes and cause genomic rearrangements to drive diverse cancers [17–20].

In light of their substantial contribution to human genetic variation and disease, it is essential that we catalogue the TE subfamilies that have been recently mobile in extant human populations, as well as the polymorphisms created by their genomic insertions. One immediate benefit of such a catalogue of recently mobile subfamilies (RMSs) would be the ability to perform targeted sequencing-based screens for causative TE insertions in diseases of unknown aetiology [21–24]. It is also important to characterize the extent to which TE subfamilies contribute to somatic genome alterations in cancer. The ultimate objective in this case would be to develop screens for oncogenic somatic insertions [25], which could facilitate the development of novel cancer therapies. For example, one could envisage the use of CRISPRi [26] to downregulate oncogenic transcripts driven by somatically inserted TE promoters [6]. Identifying the mobile subset of TEs would also help in mapping human population history [27, 28] and further our understanding of the co-evolution of host control mechanisms and mobile TEs.

The earliest RMS catalogues derive from studies of human TE insertions lacking a chimpanzee ortholog [29–32]. This approach is useful in identifying TE subfamilies that generated human-specific insertions over the last ~ 6 million years. However, the strategy appears to have limited accuracy, given that it detects TEs from numerous subfamilies thought to have become extinct before the last common ancestor of human and chimpanzee. For example, the most recent such list includes HERV-E, HERV-9, a large number of solo LTRs, the mammal-specific subfamilies *MIR* and *MIR3*, multiple anthropoid primate-specific DNA transposon families, and multiple mammalian L1 subfamilies [31]. Moreover, as described above, our interest is to identify the subfamilies that were mobile even more recently, i.e. during the divergence of human populations.

Although we are not aware of any subsequent attempts at discovering the set of human RMSs de novo, a general consensus has nevertheless emerged that the *L1Hs* subfamily, subfamilies homologous to *AluY* and the younger SVA subfamilies have recently been mobile [33]. Consequently, the focus has shifted to hypothesis-driven studies that seek to discover all human polymorphisms created by insertion of TEs from these subfamilies. Studies of this nature involve targeted sequencing of a small set of candidate RMSs in multiple individuals [34–36] or, alternatively, whole-genome sequencing (WGS) followed by annotation of polymorphic indels that match the candidate RMSs [18, 37–42]. It is likely that such studies will benefit from an updated list of known RMSs.

Due to the lack of an unbiased, high-confidence RMS list, it is currently not straightforward to distinguish between polymorphic mobile element insertions (pMEIs), which are polymorphisms created by *new TE insertions*, and polymorphic mobile element deletions (pMEDs), which are polymorphisms created by *deletion of pre-existing, ancestral TE instances* (see below). Given that there are 4,745,258 annotated TE instances covering 48% in the human genome, the likelihood of deletion of a pre-existing TE instance is high. Consequently, pMEDs are likely to be common. However, pMEIs have a more well documented role in disease causation than pMEDs [12–14, 22–24]. Moreover, pMEIs also serve as highly informative markers of human population history [27, 28]. Thus, it is important to specifically identify pMEIs in the human genome.

In this study, we present a statistical test that uses the unique indel signature of pMEIs to identify high-confidence RMSs de novo. We apply the test to 19 publicly available structural variant datasets from both long and short read WGS and thereby assign a statistical significance to each repeat subfamily annotated in the human genome by RepeatMasker [1]. TE subfamilies passing the false-discovery rate threshold are defined as putative RMSs, and the resulting RMS list is corroborated using known disease-associated TEs. Based on the catalogue of predicted pMEIs from RMSs, we examine the extent to which they may have modified functional units such as exons and promoters and functionally test one such candidate for effect on gene expression using CRISPR/Cas9 deletion. Recent TE insertions are generally refractory to regulatory genomics assays based on short-read sequencing such as ChIP-seq, DNase-seq and ATAC-seq, due to ambiguities in read mapping [43]. We therefore use an established supervised learning approach [44, 45] to bioinformatically infer the regulatory potential of pMEIs in a range of cell types. We present the entire catalogue of pMEI subfamilies, along with their predicted insertion polymorphisms and cell type specificity, as a resource for research in human disease genetics and population history. A summary of the analysis methodology is included in Supplementary Fig. 1, alongside a glossary of key terms.

## Results

### Indel signature of pMEI subfamilies

**Fig. 1:**
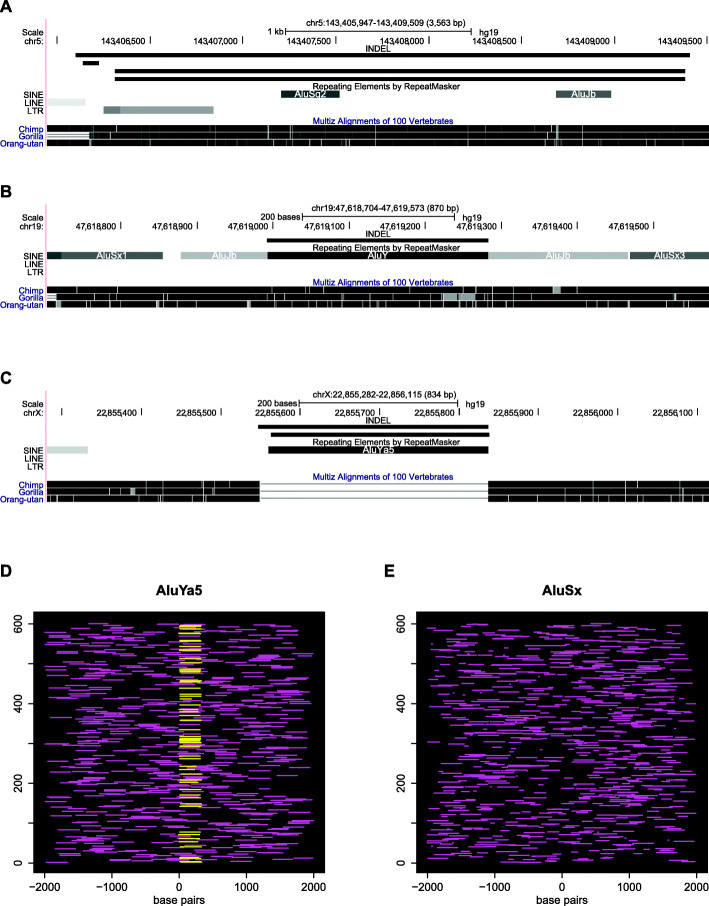
**A-C** UCSC Genome Browser screenshots of three polymorphic TE instances, hg19 assembly. INDEL track: genome-wide set of 111,509 polymorphic indels identified in 19 whole-genome sequencing datasets. Nonhuman primate sequence alignments are shown below the RepeatMasker track. **D** Location of repeat instances belonging to the recently mobile *AluYa5* TE subfamily (horizontal lines) relative to the left edges of 600 randomly chosen indels of size ≥50 bp. Yellow: *AluYa5* elements that match (≥90% mutual overlap) an indel; maroon: all other *AluYa5* elements within 2 kb of the edge of the indel. Only indels with at least one flanking *AluYa5* are included. **E** Same as **D**, for the older TE subfamily *AluSx*

We first constructed a catalogue of indel polymorphism call sets from multiple sources (Methods), so as to comprehensively identify pMEIs and pMEDs genome-wide. To illustrate differences between pMEIs and pMEDs, we first examine an *AluSq2* locus that had been reported [46] as polymorphic based on a catalogue of indels compiled from multiple sources. As can be seen from Fig. 1A, the three indels overlapping the TE all include substantial amounts of flanking sequence. In other words, the boundaries of the indels do not coincide with those of the *AluSq2* element. In contrast, we would have expected the insertion of a new *Alu* element in this locus (pMEI) to create an indel of the same size as the *Alu* sequence, with matching boundaries [47]. Thus, the observed indel pattern is more consistent with deletion of a pre-existing *Alu* element (pMED) than with insertion of a new Alu (pMEI). Moreover, the sequence alignment indicates that the *AluSq2* element is conserved in multiple other primate genomes and is thus ancestral to human. Indeed, this sequence has been annotated as a pMED [46].

A polymorphic *AluY* sequence on chromosome 19 displays a more intriguing pattern (Fig. 1B). In this case, the indel coincides almost exactly with the TE (well within the margin of error of structural variant callers), thus providing evidence in favour of a pMEI. Indeed, this locus was annotated as a pMEI in recent genome-wide survey [39]. However, the human *AluY* element is syntenically aligned in the other great ape genomes, indicating that it is actually ancestral. Thus, this is not likely to represent a true pMEI. Rather, the genome alignment indicates that a pre-existing *AluY* TE instance was polymorphically deleted, and the boundaries of the deletion coincided by pure chance with those of the TE. In other words, although the indel coincides almost perfectly with the TE, this is an example of polymorphic pMED, rather than pMEI. Chance overlaps of this kind are only to be expected, given that the human genome contains over 4.7 million TE sequences and over 100,000 structural variants have been detected in the 19 datasets we analysed in this study (Supplementary Table 1). In summary, TE-indel overlap with matching boundaries is not sufficient on its own to distinguish pMEIs from pMEDs.

An *AluYa5* polymorphism on chromosome X [47] provides an example of a more complete pMEI signature (Fig. 1C). In this case, two indels have boundaries that coincide closely with those of the TE and none of the aligned primate genomes contain an orthologous element, indicating that the TE was inserted subsequent to the divergence of the human and chimpanzee lineages. The conclusion that this most likely represents a pMEI rather than a pMED is also consistent with extensive prior evidence that *AluYa5* repeats have been highly mobile in the human population [48–50]. In light of the above, we define a putative pMEI as a TE sequence that (a) has no chimpanzee or other primate orthologues and (b) has matching boundaries (≥90% mutual overlap) with those of at least one polymorphic indel.

Despite the two filters described above, it is nevertheless conceivable that a fraction of putative pMEIs could in reality be pMEDs. For example, a TE could have inserted into the human genome after the divergence from chimpanzee (but before the common ancestor of modern humans) and then subsequently deleted with ≥90% overlap. Indeed, one previous study has suggested that pre-existing TEs could be precisely deleted through recombination between their flanking target-site duplications [51] (TSDs). However, upon manual examination, only 8 of the 36 precise human- and chimpanzee-specific TE deletions annotated in the corresponding genomes by this study appeared to be genuine, and only 2 of these were human-specific. Based on updated genome sequence alignments on the UCSC Genome Browser, the rest were resolved as TE insertions specific to human or chimpanzee (Supplementary Table 2). Thus, it appears that precise deletion of TEs is exceedingly rare, perhaps because TSDs are in most cases only 10-20 bp long and separated by hundreds of base pairs. In fact, it is not clear if such precise deletions occur with frequency greater than expected under a null model of randomly located deletions. We therefore devised a novel statistical test to determine whether any particular TE subfamily contained more putative pMEIs than expected by chance under the latter model. Subfamilies that pass this statistical test are likely to have been recently mobile.

**Fig. 2:**
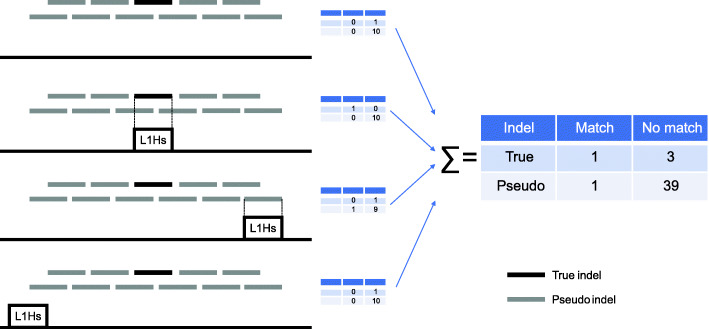
Schematic illustration of the indel-matching statistical test used to identify potential RMSs. The test is applied to one TE subfamily at a time. For each true indel (black lines), 500 flanking pseudo-indels of the same size as the true indel are simulated. For ease of illustration, true indels are all shown to be of the same size and only 10 pseudo-indels are shown (grey lines). Hypothetical locations of human-specific *L1Hs* instances in the reference genome are shown as boxes. The contingency table (Fisher’s exact test) corresponding to this toy example is constructed by summing over all true indels in the genome, as shown on the right

The intuition behind the statistical test can be illustrated by considering two TE subfamilies at opposite ends of the pMEI frequency spectrum: *AluYa5* and *AluSx*. A substantial fraction of polymorphic indels show precise overlap with human-specific *AluYa5* instances in the reference genome, and thus qualify as putative pMEIs for this subfamily (Fig. 1D). Indeed, *AluYa5* is known to have been recently active in human populations [29]. In contrast, there are no polymorphic indels forming putative pMEIs by matching human-specific TEs from the older *AluSx* subfamily, which is less likely to have created pMEIs [49] (Fig. 1E). Clearly, *AluYa5* appears to be enriched for putative pMEIs relative to *AluSx*. However, to quantify this intuition, we need a formal statistical test of the hypothesis that *AluYa5* forms more putative pMEIs than expected by chance. We therefore estimate the probability of chance matches between an indel and a human-specific *AluYa5* instance by generating 500 “pseudo-indels” (simulated indels) flanking each genuine polymorphic indel, and then counting their matches to human-specific TEs. We then use Fisher’s exact test to quantify the enrichment of actual matches relative to simulated matches **(**Fig. 2). Note that this method only considers indel polymorphisms that are deletions relative to the human reference genome, since the human-specificity of insertions cannot directly be ascertained from the whole-genome alignment on the UCSC Genome Browser.

### Genomic landscape of pMEI subfamilies

To identify TE subfamilies annotated by RepeatMasker that were likely to have contributed pMEIs to the human genome, we first excluded the subfamilies that had no human-specific instances (Methods) and then applied the above-described statistical test to each of the 464 remaining subfamilies (Supplementary Table 3). In total, only 20 TE subfamilies showed a significant association (FDR *Q* ≤ 0.001) with the indel overlap signature of pMEIs (Table 1**)**. As expected, these subfamilies are relatively “young,” with a median divergence of 0.02 substitutions/site relative to the consensus sequence. We designated these 20 subfamilies, which are likely to have spawned pMEIs in the human genome, as putative RMSs.

**Table 1:** Recently mobile subfamilies (*Q* ≤ 1e-3) and their disease associations

Row number	TE subfamily	Avg. divergence (%)	Human specific of instances	True indel matches (pMEIs)	Expected indel matches	Fold enrichment	FDR (***Q***-value)	Disease pMEI^a^ (germline)	Tumour pMEI^b^ (somatic)
1	*AluY*	3.1	1986	259	0.63	412	~ 0	7	28
2	*AluYb8*	1.3	2205	415	0.98	424	~ 0	14	22
3	*AluYa5*	0.9	3091	705	1.56	451	~ 0	25	35
4	*L1HS*	0.9	1180	142	0.68	210	5.4E-257	25	10,544
5	*AluYb9*	0.9	246	61	0.13	459	2.9E-126	5	23
6	*AluYf4*	2.1	274	53	0.12	446	2.8E-109		2
7	*AluYg6*	1.3	378	49	0.11	450	3.1E-101		4
8	*AluYc*	4.6	196	21	0.06	372	5.1E-42	4	5
9	*AluYa8*	1.6	111	19	0.04	496	1.0E-39		5
10	*SVA_F*	5.9	516	15	0.12	128	2.2E-24	6	11
11	*AluYd8*	1.1	152	11	0.03	321	1.9E-21		
12	*AluYk12*	1.2	73	10	0.02	413	2.7E-20		
13	*AluYk11*	1.8	71	9	0.03	319	1.5E-17		
14	*L1PA2*	1.8	2157	16	0.54	29	8.9E-17		
15	*LTR5_Hs*	2.2	142	9	0.04	223	1.6E-16		
16	*SVA_E*	5.0	191	8	0.04	198	2.3E-14	6	6
17	*AluYh9*	4.9	24	6	0.02	298	1.4E-11		1
18	*SVA_D*	4.1	980	7	0.20	35	7.0E-08		5
19	*L1P1*	3.6	266	3	0.04	71	3.9E-04		
20	*AluYk4*	4.3	34	2	0.00	496	5.6E-04		

^a^ Hancks, D. C., & Kazazian, H. H. (2016). Roles for retrotransposon insertions in human disease. *Mobile DNA*, *7*(1), 9

^b^Rodriguez-Martin, B. *et.al.* (2020). Pan-cancer analysis of whole genomes identifies driver rearrangements promoted by LINE-1 retrotransposition. *Nature Genetics*, *52*(3), 306–319

We hypothesized that pMEIs may have been primarily responsible for rare genetic disorders caused by de novo TE insertions. To test this hypothesis, we examined a set of 92 pMEIs implicated in rare disorders, which had been assigned to 10 subfamilies [12]. Note that there is no overlap between these rare disorder-associated TEs, which are insertions relative to the reference genome, and the indels considered in our statistical test, which are deletions relative to the reference. Surprisingly, one of the 92 pMEIs belonged to the *AluJ* subfamily, which was active ~ 65 Mya and is thought to be extinct [52]. This TE instance had been flagged as a potential recombination event between two ancestral flanking *AluJ* sequences, suggesting that it may not represent a true pMEI [53]. Another disease-causing pMEI belonged to *AluSq2*, which is again relatively ancient (> 35 Mya). *AluS*-related subfamilies have been noted for their anomalous mobility profile: though they peaked in mobility 35–60 Mya, they may nevertheless have generated a handful of recent insertions [46]. These two exceptions notwithstanding, it is remarkable that 8 of the 10 disease-causing TE subfamilies belonged to the RMS list (*P* = 1.1e-10; Fisher’s exact test, Table 1, Supplementary Table 3), and these 8 subfamilies accounted for 90 of the 92 disease-causing pMEIs (98%).

Although the 20 RMSs were identified based on germline polymorphisms, we hypothesized that some of the RMS subfamilies might also be somatically mobile, particularly in cancer cells. We therefore examined a database of 10,675 somatically inserted TEs detected using whole-genome sequencing of diverse cancers [20]. Again, this somatic TE set had no overlap with the indels we used to define RMSs. The vast majority of somatically inserted TEs in tumours belonged to subgroups of *L1Hs* (pre-Ta, Ta, Ta0, Ta1), which is the top-ranked L1 subfamily in the RMS set. Of the remaining 153 somatically inserted TEs, 147 (96%) originated from RMSs. In total, the 20 RMSs accounted for 13/17 subfamilies mobilized in tumours (*P* = 2.8e-17; Fisher’s exact test, Table 1, Supplementary Table 3). In summary, although the 20 RMSs represent only a small subset of the 934 TE subfamilies annotated by RepeatMasker, they appear to be responsible for the vast majority of TEs associated with rare genetic disorders and somatic variation in cancer.

It has been suggested that HERV repeats, which are endogenous retroviruses in the human genome, are no longer capable of transposition [54, 55]. However, an intact HERV-K provirus has been identified in a single individual with the potential for retained infectivity and a small number of HERV-K loci have shown evidence of polymorphic insertion [56, 57]. Although our analysis did not detect HERV-K per se, it did flag *LTR5_Hs*, the solo LTR created by recombination between the two near-identical LTR sequences at the flanks of *HERV-K* repeats, as a highly significant RMS (FDR *Q*-value = 1.6e-16; Table 1). Thus, our results provide statistical support to previous anecdotal reports that HERV-K has indeed been recently mobile.

**Fig. 3:**
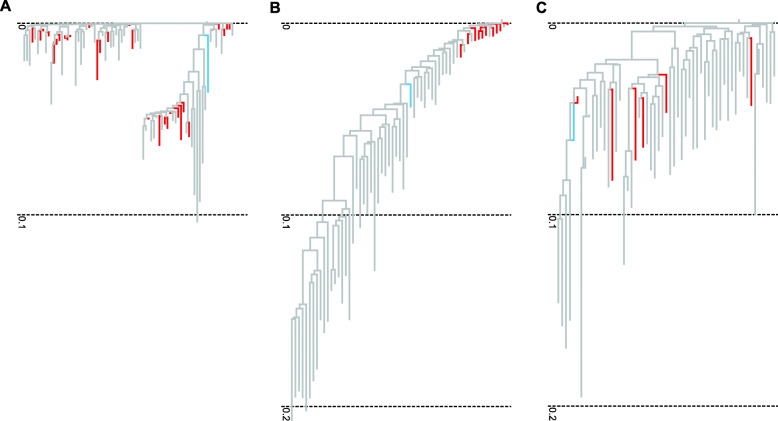
Phylogenetic relationships between pMEIs (red branches): **A** 30 randomly selected *AluYa5* pMEIs **B** the 10 *LTR5_Hs* pMEIs and **C** the 6 *SVA_D* pMEIs with length > 1000 bp. For reference, 50 randomly selected non-polymorphic MEIs from each subfamily are also shown (grey branches)

To further investigate the apparently recent mobilization of HERV-K, we examined the phylogenetic relationships of the 9 *LTR5_Hs* pMEIs identified above, as well as one additional such element detected in an expanded whole genome scan that included overlapping indels (Methods, Supplementary Table 4). For comparison, we also examined *AluYa5* (*Q* = ~ 0), a well-studied RMS that accounts for the largest number of pMEIs in our whole genome screen (Table 1, Fig. 3). The *AluYa5* pMEIs were interspersed with non-polymorphic MEIs, suggesting the existence of multiple *AluYa5* source elements (“parent” sequences) in the human genome. In contrast, *LTR5_Hs* pMEIs were very closely related, almost forming a clade within the larger *LTR5_Hs* tree. This result is consistent with a model in which most *LTR5_Hs* pMEIs arose from a single HERV-K source element. We reconstructed the consensus LTR sequence of this putative source (Supplementary Table 5) and identified a full-length HERV-K element at chr11: 101574292–101,566,761 (hg19 assembly) as its closest match in the human genome.

We also found significant evidence of recent mobility for three members of the youngest retroelement family, SVA (SINE-VNTR-Alu). The most recent SVA subfamily, *SVA_F* is already known to have generated pMEIs and the same is true for *SVA_E*, the other human-specific SVA subfamily [58]. The *SVA_D* subfamily is thought to be older, since some *SVA_D* sequences are also present in the chimpanzee and gorilla genomes. However, the majority of *SVA_D* sequences in the human genome are human-specific, and our results suggest that this subfamily has also generated pMEIs (FDR *Q*-value = 7e-8; Table 1). As above, we further examined phylogenetic relationships between *SVA_D* pMEIs and found that they were interspersed with non-polymorphic MEIs, suggesting the existence of multiple source elements.

### Chromatin openness of RMSs

Since the RMSs are relatively young, their internal promoters may have retained some ability to bind transcription factors and create regions of open chromatin. However, assays for detecting chromatin openness such as DNase-seq and ATAC-seq are not optimal for detecting regulatory elements in highly homologous repeats, due to low read mappability [43, 59]. We therefore chose to bioinformatically predict chromatin openness based on kmers present in the DNA sequence. Specifically, we used a previously validated approach [44, 45, 60] to train gapped kmer support vector machine (gkmSVM) models on DNaseI hypersensitive (DHS) sites identified in 125 human cell lines by the ENCODE consortium [61], and then used the resulting 125 models to predict hypersensitivity in each cell line at TE loci. For each cell line, the gkmSVM score threshold was chosen so that the number of predicted hypersensitive sites in the genome matched the number of measured hypersensitive sites.

**Fig. 4:**
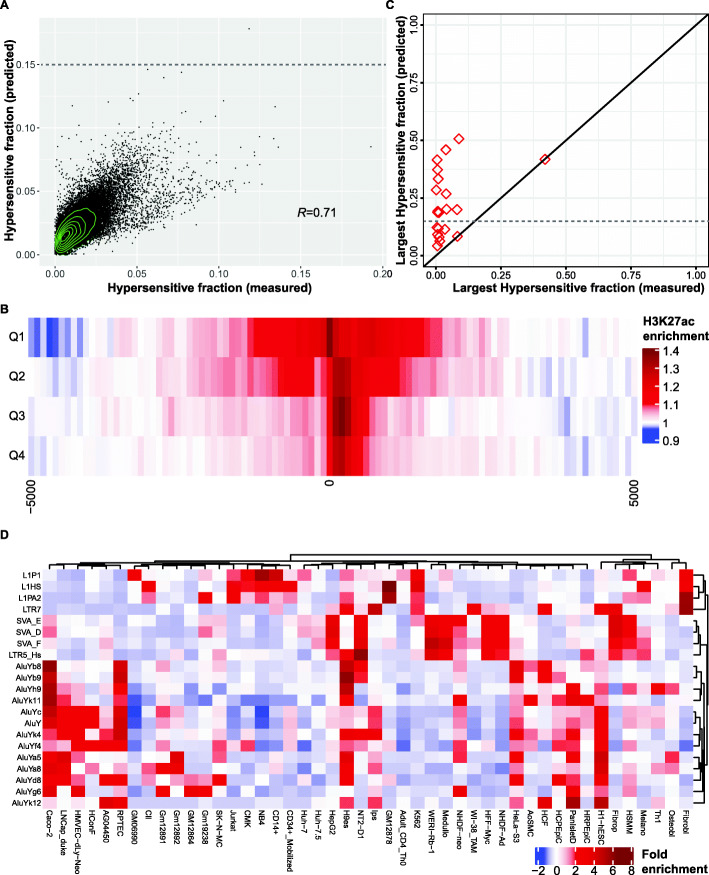
**A** Scatter-plot of measured vs. predicted hypersensitive fraction for 224 ancient TE subfamilies across 125 cell lines (28,000 points), with Pearson correlation. Dashed line: predicted fraction = 0.15. **B** Heatmap of average H3K27ac enrichment relative to flanking regions in H9 embryonic stem cells at LTR TEs genome-wide, stratified by predicted DNaseI hypersensitivity quartile. **C)** Scatter-plot of the largest hypersensitive fraction across 125 cell lines for each of the 20 RMSs: measured vs. predicted. Dashed line: predicted fraction = 0.15. **D** Heatmap of fold enrichment of predicted hypersensitive fraction for 20 RMSs plus *LTR7*. Only cell lines for which at least one of the 22 TE subfamilies has two-fold enrichment are shown

To confirm the predictive power of the gkmSVM approach, we first examined 224 ancient TE subfamilies (≥20% average divergence; ≥1000 genomic instances) in 125 cell lines. Being relatively ancient, these subfamilies are unlikely to be systematically affected by low read mappability. For each combination of subfamily and cell line, we quantified the fraction of TE instances that overlapped a DNaseI hypersensitive site by at least 50 bp. We observed that predicted and experimentally measured hypersensitive fractions were well correlated (Pearson *R* = 0.71; *P* ~  0; Fig. 4A). We also examined TEs from the LTR family, which are frequently marked by H3 lysine 27 acetylation (H3K27ac), a signature of active enhancers and promoters [59, 62–64], and found that LTRs in the higher predicted hypersensitivity quartiles showed broader domains of H3K27ac enrichment (Fig. 4B). Having confirmed the accuracy of the gkmSVM method at the subfamily level, we used it to examine the chromatin accessibility of RMSs. For each RMS subfamily, we calculated the fraction of genomic instances that were DNaseI hypersensitive in each of the 125 cell lines, and then identified the largest of these 125 hypersensitive fractions. In most cases, the largest measured hypersensitive fraction of RMSs was substantially lower than their largest predicted fraction, indicating a strong effect of low read mappability on DNase-seq data (Fig. 4C). However, *LTR5_Hs* was a strong outlier in this analysis: the measured and predicted hypersensitive fractions were both equally large (42%) for this RMS, perhaps because only a small fraction of *LTR5_Hs* TE instances were inserted recently. Notably, the largest predicted hypersensitive fraction exceeded 15% for 13/20 RMSs, as opposed to only 1/224 ancient subfamilies, indicating that RMSs are significantly enriched in open chromatin (*P* = 2e-15; Fisher’s exact test).

We then explored the cell type specificity of predicted chromatin accessibility at TEs arising from RMSs, using *LTR7* as a reference subfamily with known regulatory activity in H1 and H9 in pluripotent cells [59, 65]. When RMSs were clustered by predicted hypersensitivity across 125 cell lines, we found that Alu RMSs as a whole appeared to show specificity for pluripotent cells as well as for epithelial cell lines such as RPTEC, LNCap and, most prominently Caco-2. The latter finding is consistent with previous work showing that Alu subfamilies are DNA hypomethylated in several colon suggesting systematic regulatory activation of Alu elements in colon cancer [66]. In contrast to RMSs from other families, L1 RMSs show a preference for hypersensitivity in immune cell lines. Intriguingly, the predicted cell type specificity of the *LTR5_Hs* RMS closely followed that of the SVA subfamilies, perhaps due to the fact that the SINE-R fragment of SVA repeats is homologous to *LTR5_Hs* [67].

### Overlap with transcriptional units and gene regulatory regions

In order to gain an understanding of the impact of RMSs on transcriptional units and gene regulatory regions, we examined the genomic locations of their 1940 indel-matching instances, i.e. their 1940 predicted pMEIs (Supplementary Table 4). Though the vast majority of these putative pMEIs (97.6%) lay in intronic (767) or intergenic (1126) regions, 4 loci were found to overlap the exons of annotated genes and 43 pMEIs were present in TSS-proximal regions that tend to be enriched for promoter and proximal enhancer function (− 2 kb to + 1 kb; Supplementary Table 4). As noted above, it is difficult to detect pMEI-derived gene regulatory elements using biochemical assays based on short-read sequencing such as ChIP-seq, DNase-seq and ATAC-seq, due to low read mappability in repetitive regions. Nevertheless, we found that 105 pMEIs partially overlapped or lay within 50 bp of enhancer or promoter regions defined by ENCODE using short-read assays [68]. It is possible that these pMEIs could have contributed to gene regulation by modulating enhancer activity or creating new enhancers.

**Fig. 5:**
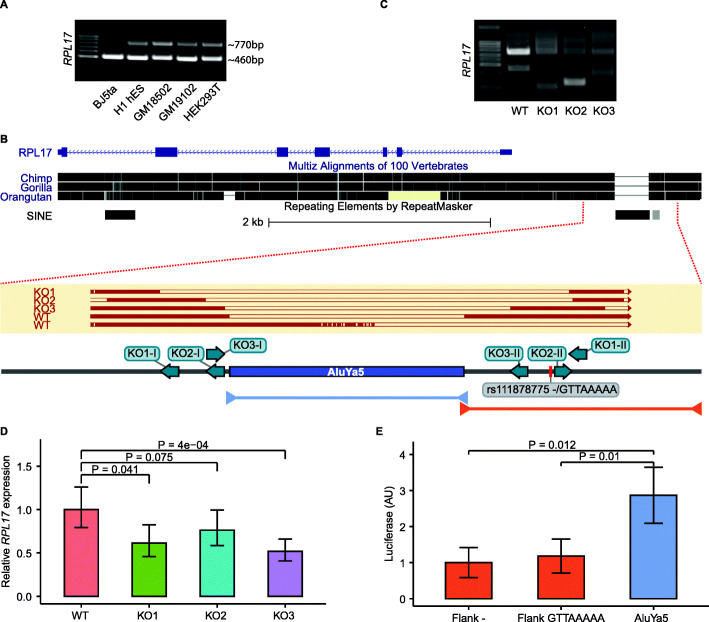
CRISPR/Cas9-mediated knockout (KO) of pMEI at the *RPL17* locus. **A)** Validation of the pMEI: PCR amplicons from genomic DNA, indicating that the *AluYa5* insertion is heterozygous in four of five tested cell lines. **B** UCSC Genome Browser track illustrating the genomic sequence context of the *AluYa5* pMEI. Alignment of Sanger sequence reads of PCR amplicons from **C)** are shown in dark red. Blue arrows indicate target sites for the three CRISPR gRNA pairs. Light blue & orange horizontal lines indicate gDNA regions used in reporter gene (luciferase) assays. **C)** PCR amplicons of wild-type (WT) HEK293T cells and three independent pooled KOs (KO1, KO2 & KO3), indicating successful KO of the *AluYa5* pMEI. **D** Relative mRNA levels of *RPL17* in WT and KO cells, as measured by qRT-PCR (*n* = 4 biological replicates). **E** Firefly luciferase levels, indicating that the *AluYa5* sequence drives substantially greater reporter gene expression than the two alleles of the size-matched flanking sequence containing the indel polymorphism rs111878775 (*n* = 5 biological replicates). Error bars indicate standard deviation

We hypothesized that pMEIs in promoter regions may have altered the expression of the corresponding genes. We therefore examined an *AluYa5* pMEI that lay immediately adjacent to a proximal enhancer like element in the promoter region of *RPL17*, a marker of poor survival in liver cancer [69] that may promote resistance to multiple chemotherapeutic drugs in gastric cancer [70]. First, to confirm that this locus represents genuine pMEI, we screened a panel of five cell lines*.* Consistently with its designation as a pMEI, this repeat instance was heterozygous in four of the five tested cell lines (Fig. 5A).

To examine the gene regulatory potential of the *AluYa5* pMEI, we used CRISPR/Cas9 to delete the abovementioned pMEI in HEK293T cells. Note that, due to the requirements of CRISPR/Cas9 guide RNA design (sequence uniqueness, presence of a PAM sequence), it was not possible to precisely excise the *AluYa5* pMEI. To minimize potential confounding effects from sequences immediately adjacent to the pMEI, we therefore performed the knockout using three independent pairs of flanking guide RNAs (Fig. 5B). This approach of testing for consistency in results from multiple pairs of guide RNAs also addresses potential off-target genomic modifications. In each case, deletion of the pMEI was confirmed by PCR amplification of the target regions, as well as Sanger sequencing of the PCR amplicons (Fig. 5B, C). Consistently, all three deletions resulted in reduced *RPL17* expression (Fig. 5D).

To further examine the regulatory potential of the *RPL17 AluYa5* element, we cloned the full length pMEI into a luciferase expression construct. As a control, we also tested both alleles of an upstream genomic sequence of similar size (Fig. 5C). Luciferase expression from the *AluYa5-*construct was approximately threefold higher than from the two controls (Fig. 5E). Together, these results indicate that the *AluYa5* pMEI may have increased the expression of *RPL17* by increasing the regulatory activity of its promoter.

To investigate the regulatory potential of pMEIs on a genome-wide scale, we analysed data from two recently published studies of allele specific expression (ASE) [71, 72]. We hypothesized that, if polymorphic MEIs were enriched for regulatory potential, their flanking genes would show an excess of ASE. Indeed, we found that the 651 expressed genes in Zhou et al. 2019 that lay within 50,000 bp of a pMEI were significantly enriched for ASE relative to the entire set of expressed genes (*P* = 1.4e-15; Fisher’s exact test; Supplementary Table 6). Similarly, the 666 expressed genes in Chen et al. 2016 that flanked a pMEI were also significantly enriched for ASE (*P* = 0.00025; Fisher’s exact test) These results indicate a significant association between pMEI proximity and ASE, which is consistent with the hypothesis that pMEIs are enriched for regulatory potential.

## Discussion

We have developed a pipeline for detecting a core set of high-confidence human RMSs and their corresponding pMEIs, based on stringent indel matching and evolutionary conservation filters (Fig. 1) and a novel statistical test (Fig. 2). The set of 20 RMSs we identified de novo using this pipeline is corroborated by highly significant overlap with known disease-causing germline pMEIs (*P* = 1.1e-10; Table 1). Intriguingly, although the RMSs were identified based on germline polymorphisms, they showed even more significant overlap with somatically mobile subfamilies in diverse cancers (*P* = 2.8e-17; Table 1). Moreover, individual RMS subfamilies had similar representation in the germline and somatic disease datasets, with one major exception: *L1Hs* elements were vastly overrepresented in the latter. These results are consistent with a model in which TE mobility is broadly similar in cancer cells and in the germline, perhaps because some genetic determinants of TE mobility, such as intact ORFs and intact protein-binding sequences (for example, SRP9/14 motifs in *Alus* [48]), are independent of cell type. Nevertheless, the great excess of *L1Hs* mobilization in cancer cells indicates that cell-type-specific mechanisms such as hypomethylation of *L1Hs* source elements in transformed cells may also play a role [19].

We compared our set of 20 RMSs to pMEI frequency-ranked subfamily catalogues from previously published studies Wang et al. 2006 (dbRIP), Stewart et al. 2011 and Gardner et al. 2017 (MELT). Although the top-ranked RMSs in these three catalogues show substantial overlap with our 20 RMSs, there are also notable differences (Supplementary Table 7). The dbRIP catalogue lists 62 RMSs, including 35 subfamilies not present in our set, due to unmatched (older) repeat names, incorrect repeat names, or failure to pass our filters. Our study includes two RMSs not present in dbRIP: *AluYk11* and *AluYk12.* Stewart et al. 2011 list 40 RMS subfamilies, including 18 not present in our set, for the same combination of reasons as listed above. Many of these are highly likely to represent false positives, for example multiple extinct *AluS* and *AluJ* subfamilies, as well as *L1P* subfamilies believed to be extinct (L1PREC, L1P3, L1P5, L1PA4–6, L1PB2, L1PBa). Manual inspection of these repeat instances revealed multiple instances of subfamily mis-annotation, imprecise overlap between the indel polymorphism and the TE and presence of primate orthologues. Our study includes three RMSs not present in Stewart et al.: *AluYk11*, *AluYk12* and *LTR5_Hs*. In contrast to the two studies discussed above, MELT uses a hypothesis-driven scan for pMEIs homologous to a predefined set of subfamilies. Nevertheless, the resulting RMS catalogue includes three likely false positives with primate orthologs: *AluYc5, Aluyf5* and *AluYc3*. Notably, 802 (27%) of the 2937 deleted *Alus* annotated by MELT as pMEIs in the hg19 reference genome had syntenic chimpanzee orthologues, suggesting that many of these sequences may actually represent pMEDs, i.e. polymorphisms created by recent deletion of ancestral TEs. Notably, the MELT catalogue contains no *SVAs* or *LTR5_Hs* elements. Overall, the above comparisons indicate that existing pMEI catalogues contain substantial false positives, false negatives and incorrect or older subfamily annotations. Our study provides an updated, high-confidence RMS list showing statistically significant overlap with indel polymorphisms. The stringent filters used to define our RMS list increase the likelihood that the corresponding 1940 indel polymorphisms represent genuine pMEIs.

Intriguingly, our RMS set includes *LTR5_Hs*, the 968 bp solo LTR created by recombination between the terminal repeats of a full-length HERV-K repeat, which ranks 15th in the RMS list (Table 1). Although some studies have identified a handful of candidate polymorphisms evidently created by very recent HERV-K insertions [38, 56, 57, 73], it is still not universally accepted that HERV-K remains active in human populations [12, 74]. Our study provides the first statistically significant evidence (*Q* = 1.6e-16) for continued insertion of HERV-K elements into human genomes. This result, in combination with the clade-restricted distribution of *LTR5_Hs* pMEIs, strongly indicates that HERV-K remains mobile and continues to contribute to human genetic diversity.

The set of 20 RMSs constitutes a resource for future studies of disease and population genetics. For example, one could discover candidate disease-causing or population-specific pMEIs by performing targeted genome sequencing using primers matching RMS consensus sequences [34–36]. Alternatively, pMEI discovery efforts based on whole-genome sequencing could use the 20 RMS consensus sequences to annotate non-reference genomic segments [18, 37–40].

A number of previous studies have noted the remarkably large contribution of TEs to gene regulatory elements and regions of open chromatin in the human genome [5–7, 9, 11, 59, 63, 64, 75–78]. However, RMSs have been mostly left out of such analyses, since they are too young for detection in genomic scans for conserved noncoding sequences, and also challenging to detect in assays based on short reads, such as DNase-seq. Using a state-of-the-art method for computationally predicting DNase hypersensitivity, we have now shown that RMSs are highly enriched for open chromatin (Fig. 4C), far more so than ancient (average divergence > 20%) repeat subfamilies. Strikingly, while fewer than 0.5% of ancient subfamilies have ≥15% hypersensitivity, 65% of RMSs exceed the same threshold. These results are consistent with an evolutionary model in which TEs are highly hypersensitive and have substantial effects on gene regulation in their “youth,” but then decay over time and recede to a low, background level of hypersensitivity as they age and their transcription factor binding sites are eroded by mutational processes [59].

In contrast to the results from this study, which shed light on the effect of TE insertion on chromatin openness, two previous studies using artificial expression of L1 retrotransposons in cell lines to infer the converse, i.e. the effect of chromatin openness on TE insertion [79, 80]. Notably, these studies found that the likelihood of L1 insertion at a genomic locus was largely insensitive to local chromatin openness or histone modifications.

In light of their strong chromatin openness, it is possible that the pMEIs created by RMSs could contribute to population variation in human gene expression. Indeed, the *AluYa5* pMEI that we tested using CRISPR/Cas9 deletion and a reporter gene assay showed a two- to threefold effect on the expression level of the flanking *RPL17* gene. Higher expression levels of *RPL17* have been associated with reduced survival in liver cancer [69]. Moreover, *RPL17* was found to be upregulated in a drug-resistant gastric cancer cell line, and overexpression of this gene conferred protection against multiple chemotherapeutic drugs in vitro [70]. It is thus intriguing to hypothesize that this *AluYa5*-derived pMEI may also have phenotypic impact, via its effect on *RPL17* expression. Furthermore, analysis of genome wide ASE datasets demonstrated significant enrichment of pMEIs in ASE loci, thus providing additional support for their potential regulatory role.

## Conclusion

Our results define a high confidence catalogue of TE RMSs. We show that these RMSs are enriched in open chromatin using suggesting that they could play a regulatory role. Using CRISPR/Cas9 deletion in vitro we demonstrate an example of an RMS pMEI that may act as an enhancer of a gene - *RPL17*. Our analysis of ASE provides further evidence of the regulatory potential of RMSs. The RMSs and pMEIs defined in this study can serve as a rich resource for future investigations into TE mobility, human population history, gene regulatory variation, germline genetic disorders and somatic mutations in tumours.

## Methods

### Identifying human-specific TEs

Since pMEIs are human-specific by definition, we first identified the set of all human-specific TEs in the human genome. TE coordinates from RepeatMasker [1] hg19 annotations (http://www.repeatmasker.org/genomes/hg19/RepeatMasker-rm405-db20140131/hg19.fa.out.gz) were mapped to five other primate genomes (PanTro5, GorGor3, PonAbe2, NomLeu3 and RheMac8) using the LiftOver tool [81] on the UCSC Genome Browser. Human-specific TEs were then defined as those having no more than 20% overlap with any of the non-human genomes. Since our analysis was focused on TE insertions, we identified human-specific TEs completely contained within tandem repeats defined by the UCSC Table Browser [82] and discarded them as potential duplications.

### Statistical test for RMSs

To compile an unbiased catalogue of indel polymorphisms in the human genome, we excluded datasets that only listed TE-matching indels, which resulted in a final dataset drawn from 19 sources (Supplementary Table 1). Since most of the polymorphic indel databases we accessed were based on the hg19 (GRCh37) assembly of the human genome, we used hg19 coordinates throughout our analysis. In the aggregated indel catalogue, we discarded polymorphic indels that were insertions relative to the reference genome, since their sequences were unavailable in many cases and therefore could not be mapped to TE subfamilies. We also discarded indels that were less than 50 bp long, since such indels are not likely to correspond to pMEIs. We then noticed multiple instances where a single TE matched the boundaries of more than one polymorphic indel (≥90% mutual overlap), due to the frequent presence of indels with near-identical boundaries. We therefore pruned the indel list to resolve overlaps. We first created an overlap graph with indels as nodes and edges connecting pairs of indels with ≥50% overlap. The nodes were then sorted by their degree (number of edges), in descending order. The first node was then removed, and the degree of each connected node was updated. Nodes were sorted again, and the entire process was repeated until all remaining nodes had zero edges. This resulted in a final set of 111,509 polymorphic indels.

To determine whether or not human-specific TE instances from a subfamily matched polymorphic indels more often than expected by chance, we first complemented each of the 111,509 true polymorphic indels in our catalogue with 500 flanking pseudo-indels (simulated indels) of the same size as the corresponding true indel. Pseudo-indels were tiled so as to overlap by 50% with their neighbours (Fig. 2). A TE instance was defined as a match to an indel if the two genomic segments showed ≥90% mutual overlap. For each subfamily, we calculated *T*_*TE*_, the number of true polymorphic indels matching a human-specific TE instance, as well as *P*_*TE*_, the number of pseudo-indels with matches. Our null hypothesis was that the indels were not caused by TE insertion, and they were therefore as likely to match any specific pseudo-indel, as they were to overlap a true indel. We therefore used Fisher’s exact test to determine if true polymorphic indels had an excess of human-specific TE matches relative to pseudo-indels. This test was performed on the 464 TE subfamilies with at least one human-specific instance. We then corrected for multiple testing by using the Benjamini-Hochberg method to estimate the false-discovery rate (*FDR* Q-value). At a Q-value threshold of 0.001, we identified 20 statistically significant RMSs (Supplementary Table 3).

### Contribution of RMSs to disease-associated MEIs

We obtained a list of 124 disease-causing germline MEIs from a previous compilation [12], of which 32 were excluded because they did not have an annotated TE subfamily. One of the 92 remaining MEIs was annotated as *AluYk13*, a subfamily with no annotated instances in the hg19 genome assembly. We therefore used the BLAT tool on the UCSC genome browser [83] to align the published sequence [84] as well as the *AluYk13* consensus sequence [85] to hg19, and both sequences perfectly matched multiple genomic regions annotated as *AluY*. We therefore re-annotated the *AluYk13* MEI as *AluY*, resulting in a total of 10 disease-causing germline MEI subfamilies (Supplementary Table 8).

Our list of TEs mobilized in cancer samples was drawn from a recent pan-cancer analysis of whole genomes [20]. This included five TE subfamilies annotated not contained in our RepeatMasker hg19 genome annotation data set: *AluYb11, AluYe5, AluYi6, AluYk2* and *AluYk3*. As above, we aligned the consensus sequences [85] for these subfamilies to hg19 using the BLAT tool. All five consensus sequences showed perfect full-length matches to hg19 sequences annotated to other TE subfamilies. Based on the RepeatMasker annotations of the aligned regions, we remapped these five subfamilies as follows: *AluYb11 > AluYb9*, *AluYe5 > AluYf4, AluYi6 > AluY, AluYk2 > AluY, AluYk3 > AluY*, resulting in a set of 17 TE subfamilies mobilized in tumours (Supplementary Table 8).

### Predicting hypersensitivity from sequence

To train a computational model of DNaseI hypersensitivity, we used a comprehensive database of 150 bp hypersensitive sites in 125 cell lines (ENCODE “Master” list, January 2011 freeze). Each hypersensitive site was extended by one base pair to the left, for compatibility with the workflow below. For each of the 125 cell lines, we used LS-GKM, a large-scale implementation of the gkmSVM method [44], to train a model of regulatory sequences using the corresponding set of ENCODE hypersensitive sites as the positive set, and random genomic sequences of equal size as the negative set. The positive and negative sequence sets were matched for GC content and only sequences that showed no overlap with TEs were included in the training. The size of the positive set was fixed at 70,000 for each cell type. We used default LS-GKM parameters for training with *t* = 4, *l* = 11, *k* = 7, d = *3*, *c* = 1, *e* = 0.001. A total of 125 cell-type-specific models of DNaseI hypersensitivity were thus constructed, one for each cell line.

To predict DNaseI hypersensitivity genome-wide in each cell line, we tiled hg19 into 151 bp regions with 76 bp overlap between consecutive tiles, resulting in ~ 38 million tiles, each of which was assigned a gkmSVM hypersensitivity score. For each cell line, overlapping 151 bp segments with gkmSVM score ≥ 1 were merged up to a maximal size of 601 bp, assigned the score of their best-scoring segment and then resized to a 151 bp region by trimming the edges symmetrically. All resulting genomic regions were then ranked by their gkmSVM score and a score threshold was selected so that the final number of regions predicted to be hypersensitive was the same as the number of measured hypersensitive sites in the same cell type.

### Hypersensitivity of RMSs

Analysis of the DNaseI hypersensitivity (chromatin openness) of a TE subfamily was based on the entire set of hg19 TE instances belonging to the subfamily, including those that were not annotated as polymorphic. A TE instance was defined as DNaseI hypersensitive in a cell line if it overlapped a hypersensitive site by ≥50 bp. The hypersensitive fraction of each TE subfamily in each cell line was defined as the ratio of hypersensitive instances to total instances in the genome. In this manner, we computed 28,000 (125 × 224) hypersensitive fractions for the 224 ancient TE subfamilies (Fig. 4A). The largest hypersensitive fraction of a TE subfamily was defined as the maximum value of its hypersensitive fraction across the 125 cell lines (Fig. 4C). Cell type specificity: for each TE subfamily, the hypersensitivity fold enrichment was calculated as the ratio of the hypersensitive fraction in any given cell line to the average hypersensitive fraction across all 125 cell lines (Fig. 4D).

### Overlap of pMEIs with transcriptional units

As noted above, indel match statistics for individual TE subfamilies were calculated based on unique (≤50% mutual overlap) indels, which yielded a final tally of 1820 pMEIs (Table 1). However, this tally potentially leaves out pMEIs that could be inferred from the overlapping, discarded indels. To generate a more complete list of pMEIs, we therefore scanned for TE instances from the 20 RMSs that showed ≥90% mutual overlap with the discarded indels and added these to the dataset, resulting in a final set of 1940 putative pMEIs in the hg19 reference genome (Supplementary Table 4). We then annotated the subset with ≥5 bp overlap with an exon from the UCSC Known Gene annotation set as exonic. Of the remaining pMEIs, those that overlapped a promoter region (2 kb upstream and 1 kb downstream of a transcription start site) by ≥50 bp were defined as promoter pMEIs and the rest were defined as intronic or intergenic based on their location relative to gene bodies.

We also overlapped all the pMEIs, with the promoter like, proximal enhancer like and distal enhancer like candidate cis-regulatory elements defined by ENCODE [68]. The GRCh38 annotations were downloaded and lifted over to hg19 using UCSC lift-over tool. Due to low read mappability of pMEI sequences we extended the coordinates of the ENCODE candidate cis-regulatory elements by 50 bp both up and downstream and used these coordinates to assign the pMEI to promoter, proximal enhancer and distal enhancer elements.

### In vitro validation and CRISPR knockout

All restriction enzymes were purchased from NEB. PCR reactions were conducted using Q5® Hot Start High-Fidelity 2X Master Mix (NEB, M0494L). Ligations were conducted using isothermal assembly with NEBuilder® HiFi DNA Assembly Master Mix (NEB, E2621L). All primers & oligos were ordered from Integrated DNA Technologies, Singapore.

Genomic DNA of a panel of cell lines (Supplementary Table 9) was screened to determine presence or absence of TE polymorphism. PCR primers for screening pMEIs at the promoter region of *RPL17* are found in Supplementary Table 10.

The genomic sequence of the TE upstream of *RPL17* (chr18: 47019368–47,020,675) was uploaded to the CRISPOR server [86] (crispor.tefor.net) Single gRNAs were designed using the online search algorithm and high-scoring candidates tightly flanking the *AluYa5* insertions were selected (Supplementary Table 10). Two pairs of gRNAs flanking the TE were annealed and ligated into pMIA3 1sg-eSpCas9-2AmRuby2-2Amp53DD (Addgene plasmid #109399) [87].

The gRNA cutting was confirmed by GFP reconstitution assay using the pCAG-eGxxFP plasmid (Addgene plasmid # 50716), a gift from Masahito Ikawa, as described previously [88]. Briefly, the target sequence was amplified from HEK293T gDNA cloned into the SalI cut site on pCAG-eGxxFP (for primers see Supplementary Table 10). The resulting plasmid was transfected into HEK293T cells with or without pMIA3 + gRNA. 48 h later, strong GFP signal was observed when both plasmids were transfected, indicating Cas9 cutting activity (data not shown).

The TE was targeted in HEK293T cells with three independent sets of flanking gRNAs using Lipofectamine 3000 (L3000015, Thermo Fisher Scietific) according to manufacturer’s instructions. Approximately 48 h post-transfection, cells positive for mRuby2 expression were sorted through flow cytometry and expanded further. Successful knockout (KO) of the TE was confirmed by PCR and Sanger sequencing from gDNA samples (PureLink™ Genomic DNA Mini Kit, K182002, Thermo Fisher Scientific) using specific primers (Supplementary Table 10). RNA was extracted from three biological replicates of the pooled KO and untargeted cells (Direct-zol^Tm^ RNA Miniprep kit, ZYR.R2052, Zymo Research) and 1 μg converted into cDNA (Superscript IV Vilo MM, 11766050, Thermo Fisher Scientific). Quantitative PCR reactions using TaqMan gene expression assays and master mix were used to compare the expression levels of *RPL17* against reference genes *18S* and *GAPDH* (Hs99999901_s1 and Hs03929097_g1, and 4,444,557, Thermo Fisher Scientific). Quantitative RT-PCR analysis was done as described previously [89].

### Luciferase analysis

The 348 bp region containing the full *AluYa5* pMEI was amplified from HEK293T genomic DNA and cloned into the multiple cloning site upstream of the minimal promoter of the pGL4.23 plasmid (pGL4.23 [luc2/minP], E8411, Promega). A size-matched flanking region upstream of the *AluYa5* pMEI was amplified and cloned in a similar manner into the pGL4.23 plasmid. Primers used for amplification are listed in Supplementary Table 10. Since this flanking region contained an indel polymorphism (rs111878775), two independent plasmids were constructed, one for each allele. The three plasmids were transfected into HEK293T cells in 96-well plates using Lipofectamine 3000, as described above. Alongside the test plasmids, we also transfected a positive control plasmid, pGL4.13. All reactions also contained a plasmid expressing *Renilla*-luciferase (pGL4.73) as a transfection efficiency control. All transfections were performed in technical quadruplicates. After 48 h luciferase expression was quantified using the Dual-Glo Luciferase Assay System (E2920, Promega) according to manufacturer’s instructions. The transfections were repeated 5 times (5 biological replicates) and the normalised luciferase expression levels from these replicates were used for statistical analysis.

### Analysis of allele specific expression data

The coordinates of the nearest genes for all of the 1940 putative pMEIs in the hg19 reference genome (Supplementary Table 4) were filtered for distance< 50,000 bp from the pMEI, resulting in a list of 1081 pMEI-flanking genes (Supplementary Table 6). The lists of ASE genes as well as all genes analysed were obtained from Chen *et.al*. 2016 and Zhou *et.al.* 2019. The ASE status of the 1081 pMEI-flanking genes was determined using either dataset and Fisher’s exact test was applied to quantify the statistical significance of pMEI enrichment near ASE genes.

### Sequence alignments of pMEIs

Underlying sequences of the 10 *LTR5_Hs*, the 6 *SVA_D* greater than 1000 bp and 30 randomly selected *AluYa5* pMEIs as well as 50 randomly selected non-pMEI instances of each TE were downloaded from USCS genome browser. The above sequences were respectively collated together with the consensus sequence of each TE obtained from DFAM [90] (Supplementary Table 11) and aligned using the EMBL-EBI [91] online tool Clustal Omega [92] with default parameters. Phylogenetic trees were created using the online tool IcyTree [93].

### Manual inspection of pMEI from published catalogues

Published datasets of pMEIs [37, 39, 42] were downloaded and the coordinates for TE instances annotated as polymorphic deletions in relation to the reference genome were used for verification. Selection of instances from TE subfamilies not listed in the RMS catalogue from the current study were manually inspected on the UCSC genome browser using the hg19 assembly and RepeatMasker track as well as the multiz alignment track for chimpanzee (panTro6), gorilla (gorGor6) and orang-utan (ponAbe3). To assess ancestral status of a group of pMEIs, their coordinates were lifted over to the chimpanzee (panTro6) assembly using the UCSC LiftOver tool with 0.3 minimum ratio of bases that must remap. Successful lift over was defined as syntenic conservation in the corresponding ape genome.

## Supplementary Information

**Additional file 1: Supplementary Figure 1.** A) Summary of the datasets and analysis workflow. B) Glossary of important terms.

**Additional file 2: Supplementary Table 1.** Source datasets used for polymorphic indel dataset generation. **Supplementary Table 2.** Manual verification of pMEDs reported in van de Lagemaat et. al. (2005) & list of current assemblies used for verification on the UCSC Genome Browser. **Supplementary Table 3.** Full list of TE subfamilies with human specific instances and their associated statistics. **Supplementary Table 4.** Full list of pMEIs identified from RMSs. **Supplementary Table 5.** LTR5_Hs pMEI consensus sequence. **Supplementary Table 6.** Genes analysed for pMEI and ASE correlation. **Supplementary Table 7.** Comparison of our RMS to frequency ranked lists from published pMEI datasets. **Supplementary Table 8.** Disease associated TE subfamilies and their hg19 remapping. **Supplementary Table 9.** Cell lines used in the study. **Supplementary Table 10.** Primers and oligos used in the study. **Supplementary Table 11.** Underlying DNA sequences of pMEIs and non-pMEI TE used for alignment.

## Data Availability

All data generated during this study are included in this published article and its supplementary information files. Links to external data sources are listed below. http://www.repeatmasker.org/genomes/hg19/RepeatMasker-rm405-db20140131/hg19.fa.out.gz ftp://ftp-trace.ncbi.nlm.nih.gov/giab/ftp/data/ChineseTrio/analysis/PacBio_pbsv_10072018/HG005%2B6%2B7_GRCh37_pbsv.vcf.gz ftp://ftp-trace.ncbi.nlm.nih.gov/giab/ftp/data/AshkenazimTrio/analysis/NIST_SVs_Integration_v0.6/HG002_SVs_Tier1_v0.6.vcf.gz https://www-ncbi-nlm-nih-gov.ejproxy.a-star.edu.sg/sra/?term=SRP115881 https://www.ncbi.nlm.nih.gov/bioproject/PRJNA38505 ftp://ftp-trace.ncbi.nlm.nih.gov/giab/ftp/data/AshkenazimTrio/analysis/Baylor_sniffles_05092017/all_reads.fa.giab_h002_ngmlr-0.2.3_mapped.bam.sniffles1kb_auto_noalts.vcf.gz ftp://ftp-trace.ncbi.nlm.nih.gov/giab/ftp/data/AshkenazimTrio/analysis/Baylor_sniffles_05092017/all_reads.fa.giab_h003_ngmlr-0.2.3_mapped.bam.sniffles1kb_auto_noalts.vcf.gz ftp://ftp-trace.ncbi.nlm.nih.gov/giab/ftp/data/AshkenazimTrio/analysis/Baylor_sniffles_05092017/all_reads.fa.giab_h004_ngmlr-0.2.3_mapped.bam.sniffles1kb_auto_noalts.vcf.gz https://github.com/nanopore-wgs-consortium/NA12878/blob/master/Genome.md ftp://ftp-trace.ncbi.nlm.nih.gov/giab/ftp/data/NA12878/NA12878_PacBio_MtSinai ftp://ftp-trace.ncbi.nih.gov/giab/ftp/data/NA12878/NA12878_PacBio_MtSinai/ https://www.biorxiv.org/content/early/2017/08/10/174938 https://www.ncbi.nlm.nih.gov/dbvar/?term=nstd144 https://www.ncbi.nlm.nih.gov/bioproject/?term=PRJNA380394 https://www.ncbi.nlm.nih.gov/bioproject/?term=PRJNA481779 https://www.internationalgenome.org/phase-3-structural-variant-dataset https://www.ncbi.nlm.nih.gov/bioproject/PRJNA301527/ https://api.wenglab.org/screen_v13/fdownloads/GRCh38-ccREs.PLS.bed https://api.wenglab.org/screen_v13/fdownloads/GRCh38-ccREs.pELS.bed https://api.wenglab.org/screen_v13/fdownloads/GRCh38-ccREs.dELS.bed

## Abbreviations

TE: Transposable elements
RMSs: Recently mobile subfamilies
WGS: Whole-genome sequencing
pMEIs: Polymorphic mobile element insertions
pMEDs: Polymorphic mobile element deletions
TSDs: Target-site duplications
gkmSVM: Gapped kmer support vector machine
DHS: DNaseI hypersensitive
H3K27ac: H3 lysine 27 acetylation
ASE: Allele specific expression
KO: Knockout
NRF: National Research Foundation
