# Supplementary material for: Transposable elements that have recently been mobile in the human genome
Source: BMC Genomics. 2021 Nov 3;22:789. doi: 10.1186/s12864-021-08085-0 (PMC8567694; doi:10.1186/s12864-021-08085-0)
Supplement: Supplementary file 1 — Additional file 1: Supplementary Figure 1. A) Summary of the datasets and analysis workflow. B) Glossary of important terms. [file 12864_2021_8085_MOESM1_ESM.pdf]

A

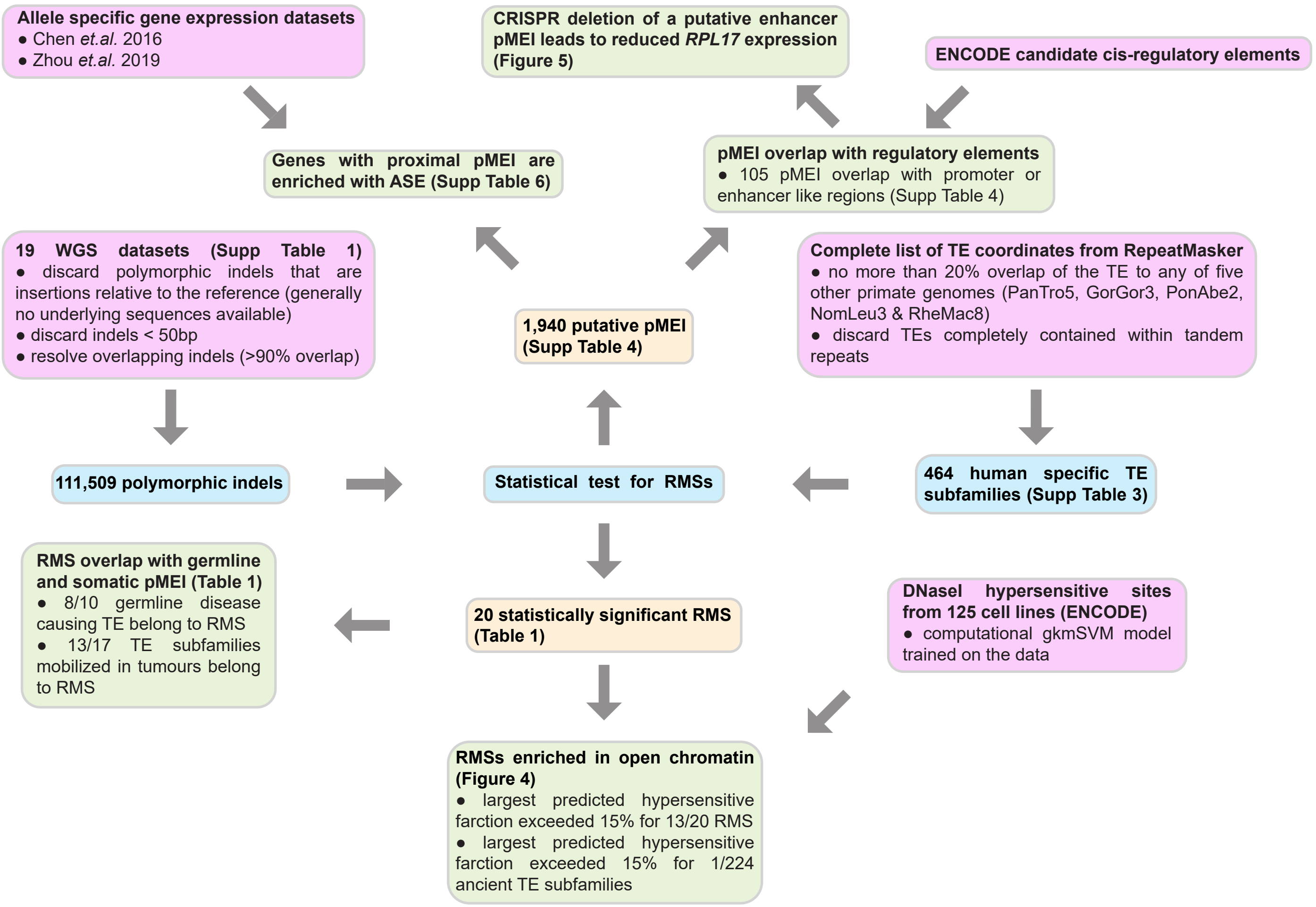

B

Glossary of important terms:

**repetitive regions**	=	regions of the genome where the underlying DNA sequence is repeated in multiple locations. TE are a subset of repetitive regions. Other examples include satellite and ribosomal DNA as well as seqmental duplications
**TE**	=	transposable elements, DNA sequences that have the ability to move (transpose) to new locations in the genome
**RMS**	=	recently mobile subfamilies, TE subfamilies that have been mobile in extant human populations
**pMEI**	=	polymorphic mobile element insertion, a TE insertion that is polymorphic within human populations and has (a) no primate orthologues and (b) matching boundaries (>90% mutual overlap) with at least one polymorphic indel
**indel**	=	a genetic mutation caused by the insertion or deletion of a segment of DNA
